# DIFFERENTIAL PATTERNS OF THE RELATIONSHIP BETWEEN EXERCISE DOSE AND MORTALITY RISK ACROSS SEVERITIES OF AIRFLOW LIMITATION: A PROSPECTIVE COHORT STUDY WITH A 5-YEAR FOLLOW-UP PERIOD

**DOI:** 10.2340/jrm.v57.43377

**Published:** 2025-06-16

**Authors:** Yide WANG, Hongxia DUAN, Yingqi WANG, Yidie BAO, Linhong JIANG, Xiaoyu HAN, Fengsen LI, Peijun LI, Weibing WU, Xiaodan LIU

**Affiliations:** 1 Department of Rehabilitation, The Fourth Clinical Medical College of Xinjiang Medical University, Urumqi, Xinjiang; 2 School of Rehabilitation Science, Shanghai University of Traditional Chinese Medicine, Shanghai; 3 Institute of Rehabilitation Medicine, Shanghai Academy of Traditional Chinese Medicine, Shanghai; 4 Engineering Research Center of Traditional Chinese Medicine Intelligent Rehabilitation, Ministry of Education, Shanghai; 5 Department of Integrated Pulmonology, Fourth Clinical Medical College of Xinjiang Medical University, Urumqi; 6 Department of Sports Rehabilitation, Shanghai University of Sport, Shanghai, China

**Keywords:** airflow limitation, mortality risk, physical activity dosage, prospective cohort

## Abstract

**Objective:**

This study examines the dose–response relationship between physical activity (PA) and all-cause mortality across different severities of airflow limitation, identifying threshold effects that yield new insights into the PA–mortality association.

**Design:**

A prospective cohort study with a 5-year follow-up (2018–2023), employing multivariate Cox models and penalized spline smoothing to assess non-linear associations.

**Subjects/Patients:**

A total of 2,975 individuals from a cohort categorized by airflow limitation severity (normal, GOLD 1–4).

**Methods:**

PA levels were quantified in metabolic equivalent hours per week (MET·h/week). Cox proportional hazards models were used to evaluate PA–mortality associations, with penalized spline analysis detecting threshold effects.

**Results:**

Identified thresholds were 41.50 MET·h/week (95% CI: 23.03–64.22) for normal lung function and 13.21 MET·h/week (95% CI: 9.67–16.14) for GOLD 1. Below these thresholds, higher PA levels were associated with a significant reduction in mortality risk (HR = 0.66, HR = 0.41, respectively). In GOLD 2, PA levels below the threshold were associated with a lower mortality risk (HR=0.85), whereas PA exceeding the threshold was associated with a higher mortality risk (HR = 1.23). No significant associations were observed in GOLD 3–4.

**Conclusion:**

PA demonstrates a non-linear, threshold-dependent association with mortality. These findings underscore the importance of individualized PA recommendations for optimizing health outcomes in individuals with chronic respiratory conditions.

## LAY ABSTRACT

Many people with chronic lung conditions struggle with breathing problems, which can affect their daily lives and overall health. Regular physical activity is known to improve lung function and reduce the risk of complications, but how much exercise is beneficial remains unclear. Our study followed a large group of individuals over 5 years to examine how different levels of physical activity impact survival rates, especially in those with varying degrees of airflow limitation. We found that moderate exercise was associated with a lower risk of death among individuals with normal lung function or mild impairment. However, too much exercise may not be beneficial for those with more severe lung conditions. These findings highlight the need for personalized exercise recommendations based on lung health. By tailoring physical activity levels to individual needs, healthcare providers can help patients improve their quality of life and reduce health risks associated with lung diseases like COPD.

Chronic obstructive pulmonary disease (COPD) represents a substantial global health burden, afflicting more than 200 million individuals and contributing significantly to disability and healthcare utilization (1). Responsible for roughly 5% of all deaths worldwide, COPD has recently risen to become the third leading cause of mortality, imposing significant economic and healthcare burdens (2–4). Current guidelines advocate for the inclusion of exercise rehabilitation as an adjunct to pharmacological treatments, given its proven benefits in enhancing respiratory function and physical health, alleviating symptoms, and reducing mortality (5–7). Physical activity (PA), a cornerstone of exercise rehabilitation, has demonstrated positive effects across various conditions including osteoarthritis, type 2 diabetes, stroke, and clinical depression (8–11). Cohort studies have consistently reported an inverse relationship between PA levels and mortality (12, 13). However, the efficacy of PA specifically within COPD populations remains contentious. While a meta-analysis supports the benefits of PA for other chronic conditions, the evidence regarding COPD is considerably weak (14); 1e study even suggests that high-intensity rehabilitation programmes may increase the mortality risk in COPD patients(15). This ambiguity underscores the need for a clearer understanding of the dose–response relationship between PA and airflow limitation-related diseases outcomes.

Furthermore, the majority of existing studies categorize participants into quantiles, which limits comparability and clinical applicability (16–18). Most research focuses on linear associations between PA and airflow limitation-related diseases such as COPD mortality, overlooking potential non-linear relationships (19). Consensus on optimal PA levels is lacking, and few studies have systematically assessed the impact of different PA dosages on COPD mortality. Additionally, given the heterogeneity of COPD, varying degrees of airflow limitation may elicit differential responses to PA. However, systematic evaluations of these variations are scarce. To address these gaps, we conducted a prospective cohort study to investigate the differential patterns of the association between exercise dosage and mortality risk among patients with varying degrees of airflow limitation and to try to identify the range of optimal exercise dose.

## METHODS

### Study population and design

This study is a branch of the Northwest Natural Population Cohort Study, a longitudinal cohort investigation covering 75% of Moyu County, Hotan Prefecture, Xinjiang, involving 102 villages across 7 towns (20). Baseline data were collected from June to December 2018 in rural areas of Northwest China’s Moyu County. Participants were followed up for 5e years using a unique identifier linked across health check-ups, medical records, chronic disease management, and death registration systems. Follow-up interviews were conducted via telephone or face-to-face meetings, prioritizing those without subsequent data to validate system information.

Participants were excluded if they were under 35 years old, lacked essential demographic data (gender, age, educational level), or had missing vital status or PA information. The Ethics Committee of the Fourth Clinical Medical College of Xinjiang Medical University approved the study protocol and adhered to the principles outlined in the Declaration of Helsinki. Written informed consent was obtained from all participants.

### Data collection

Demographic characteristics, lifestyle habits, medical histories, and PA levels were collected using structured questionnaires administered by trained field investigators. Questionnaires were developed by the Northwest Regional Natural Population Cohort Study Group and recorded on paper forms. Data were entered and verified by designated personnel within the Northwest Regional Natural Population Cohort Study Center’s data platform, which includes logic checks and quality control mechanisms.

PA was quantified by multiplying the metabolic equivalent task (MET) intensity, frequency, and duration (hours) to yield MET hours per week. MET scores were referenced according to the standards outlined in the Physical Activity Questionnaire (PAQ_E) section of the National Health and Nutrition Examination Survey (NHANES) (21, 22). Based on weekly PA volume (MET-h/week), participants were classified into 3 categories: None/Insufficient PA (< 7.5 MET-h/week), Recommended PA (7.5–15 MET-h/week), and Additional PA (> 15 MET-h/week). These categories correspond to PA levels that are below, meeting, and exceeding the standard recommended physical activity levels, respectively. Lung function was evaluated using the Masterscreen Pneumo device (version 2.13; CareFusion, Würzburg Germany). The GOLD grades airflow limitation in stable COPD by post-bronchodilator forced expiratory volume in 1 s (FEV_1_) expressed as percentage of predicted. GOLD 1: mild, FEV_1_ ≥ 80% predicted; GOLD 2: moderate, 50–79%; GOLD 3: severe, 30–49%; GOLD 4: very severe, < 30%. Body composition was measured using the TANITA DC-430MA body composition analyser (TANITA Inc., Tokyo, Japan).

### Statistical analysis

Continuous variables such as age and body fat mass were summarized as means ± standard deviations. Categorical variables, including gender, smoking status, and marital status, were presented as percentages. For continuous variables, inter-group differences were assessed with Student’s *t*-test when the data conformed to a normal distribution; when normality was not satisfied, the Mann–Whitney U test was employed.

Comparisons of categorical variables between groups were conducted using χ² tests.

To investigate the relationship between PA and mortality, Cox proportional hazards models were applied, adjusting for age and gender (Model 1), further adjusted for education, annual income, marital status, tea consumption, tobacco and alcohol use, BMI, body fat percentage, body fat mass, muscle mass, muscle rate percentage, and visceral adiposity index (Model 2). Model 3 (fully adjusted model) included all covariates from Model 2 and added the following clinically pertinent variables: lung function, chronic bronchitis, asthma, diabetes, hypertension, chronic kidney disease, rheumatoid arthritis, blood glucose, triglycerides, total cholesterol, low-density lipoprotein cholesterol, and high-density lipoprotein cholesterol. To examine the threshold effect of PA on all-cause mortality, a Cox proportional hazards regression model integrated with a generalized additive model and smooth curve fitting (using the penalized spline method) was employed. The proportional hazards assumption for each Cox model was evaluated using Schoenfeld residual tests (Survival package in R software; R Foundation for Statistical Computing, Vienna, Austria), and no violations of proportionality were detected in any model. The penalized spline approach allowed us to model the PA–mortality relationship flexibly, without assuming linearity, thereby helping to detect any non-linear association. If nonlinearity was detected, a breakpoint was calculated using a recursive algorithm, and 2-piecewise Cox proportional hazards models were constructed around the breakpoint. This 2-piecewise model provides separate hazard ratio estimates for PA below and above the identified threshold, capturing any threshold-dependent change in risk. The optimal breakpoint was determined by maximizing the model likelihood, and the significance was tested by comparing the log-likelihood ratio between 1-piece and 2-piece models. Log-transformation of MET hours per week was performed to assess the relationship with every standard deviation increase in MET hours per week and all-cause mortality.

We applied multivariate multiple imputation with chained equations to enhance statistical power and reduce bias due to missing data (23). The primary outcomes were reanalysed using these imputed datasets to verify the robustness of our findings. Additionally, subgroup analyses and interaction tests were performed to assess the moderating effects of covariates on the association between PA and all-cause mortality. Statistical analyses were performed using STATA version 14.0 (STATA Corp, College Station, TX, USA) and R software (version 3.6.3; https://www.R-project.org). A *p*-value ≤ 0.05 was considered statistically significant.

## RESULTS

### Baseline characteristics of the study cohort

In this study, 2,975 participants were enrolled, 49.63% were female, and the mean age at baseline was 51.47 ± 9.80 years (Table I). The cohort’s baseline demographics and clinical characteristics are summarized, with all-cause mortality serving as the outcome variable following a 5-year follow-up. Key risk factors for increased mortality included advanced age, male sex, high body mass index, elevated body fat percentage, increased visceral adiposity, reduced muscle mass, impaired pulmonary function, low exercise dose, and a history of tuberculosis, COPD, chronic bronchitis, hypertension, or chronic kidney disease. No significant differences were observed between the Alive and Dead groups for other variables. Comprehensive analyses were conducted, stratifying sociodemographic features and other covariates by gender (Table SI).

**Table I:** Demography and clinical characteristics of participants

Characteristics	Total (*n* = 2,975)	Alive (*n* = 2,892)	Dead (*n* = 83)	*p*-value
Age, years^*^	51.47 ± 9.80	51.23 ± 9.69	59.98 ± 9.83	<0.001
Female, %^***^	49.63	50.02	36.14	0.013
Education, %^***^				0.271
Illiterate	14.28	14.07	21.69	
Primary school	71.17	71.34	65.06	
Junior high school	12.80	12.82	12.05	
High school and above	1.76	1.77	1.20	
Gross annual income, yuan^***^				0.074
<10,000	54.23	53.95	63.86	
≥10,000	45.77	46.05	36.14	
Marital status^***^				0.083
Married	85.01	85.20	78.31	
Divorced etc	14.99	14.80	21.69	
Tea consumption^***^				0.101
Yes	52.23	52.48	43.37	
No	47.77	47.52	56.63	
Tobacco consumption^***^				0.721
Yes	13.52	13.56	12.20	
No	86.48	86.44	87.80	
Alcohol consumption^***^				0.780
Yes	9.31	9.34	8.43	
No	90.69	90.66	91.57	
BMI, kg/m^2*a^	26.93 ± 6.17	25.53 ± 4.19	26.96 ± 6.20	0.023
Body fat percentage,%^*^	31.48 ± 9.50	29.05 ± 8.31	31.54 ± 9.53	0.018
Body fat mass, kg^*^	20.87 ± 8.50	18.69 ± 6.65	20.93 ± 8.54	0.018
Muscle mass, kg^*^	41.84 ± 8.29	42.44 ± 8.50	41.82 ± 8.29	0.399
Muscle rate percentage, %^*^	0.65 ± 0.09	0.67 ± 0.08	0.65 ± 0.09	0.026
Visceral adiposity index^*^	10.34 ± 4.30	10.29 ± 4.27	11.96 ± 4.90	<0.001
Physical activity, metabolic equivalent·h/week^**b^	45.16 ± 73.71	45.38 ± 74.38	37.64 ± 43.90	0.007
FVC% predicted^*^	96.69 ± 19.36	96.90 ± 19.29	89.27 ± 20.34	<0.001
FEV1% predicted^*^	88.45 ± 20.35	88.71 ± 20.28	79.47 ± 21.07	<0.001
FEV1/FVC^*^	76.11 ± 9.66	76.19 ± 9.55	73.44 ± 12.86	0.010
Tuberculosis^***^				<0.001
No	95.02	95.77	63.49	
Yes	4.98	4.23	36.51	
Chronic obstructive pulmonary disease^***^				<0.001
No	78.76	79.18	63.86	
Yes	21.24	20.82	36.14	
Chronic bronchitis^***^				0.006
No	83.33	83.64	72.29	
Yes	16.67	16.36	27.71	
Asthma^***^				0.070
No	97.21	97.30	93.98	
Yes	2.79	2.70	6.02	
Diabetes^***^				0.066
No	96.17	96.25	89.66	
Yes	3.83	3.75	10.34	
Hypertension^***^				0.004
No	69.32	69.60	44.83	
Yes	30.68	30.40	55.17	
Chronic kidney disease^***^				0.167
No	92.97	93.08	89.16	
Yes	7.03	6.92	10.84	
Rheumatoid arthritis^***^				0.873
No	86.15	86.13	86.75	
Yes	13.85	13.87	13.25	
Blood glucose, mmol/L^**^	5.33 ± 1.49	5.32 ± 1.41	5.60 ± 3.11	0.106
Triglyceride, mmol/L^**^	1.43 ± 1.16	1.43 ± 1.15	1.63 ± 1.53	0.140
Total cholesterol, mmol/L^*^	4.63 ± 1.36	4.63 ± 1.36	4.72 ± 1.39	0.580
Low density lipoprotein, mmol/L^*^	2.58 ± 0.78	2.58 ± 0.78	2.59 ± 0.87	0.798
High density lipoprotein, mmol/L^*^	1.32 ± 0.48	1.32 ± 0.49	1.28 ± 0.39	0.483

For continuous variables, inter-group differences were assessed with Student’s *t*-test^*^ when the data conformed to a normal distribution; when normality was not satisfied, the Mann–Whitney *U* test^**^ was employed. Comparisons of categorical variables between groups were conducted using χ² tests^***^.

a Body mass index (BMI) was calculated as bodyweight in kilograms divided by the square of the height in metres.

b metabolic equivalent·h = metabolic equivalent score × exercise time. FEV1: forced expiratory volume in 1 s; FVC: forced vital capacity.

### Association of PA with all-cause mortality

The study employed 3 Cox proportional hazards models to evaluate the independent impact of PA on all-cause mortality, as outlined in Table II. Hazard ratios and their corresponding 95% confidence intervals were estimated using these models. The fully adjusted model (Model 3) included covariates such as age, gender, education level, gross annual income, marital status, tea consumption, tobacco use, alcohol intake, BMI, etc. In this model, an increment of 1 standard deviation in MET-h/week was linked to a 12% reduction in the risk of all-cause mortality (HR = 0.88; 95% CI: 0.79–0.99; *p* = 0.0169). Subgroup analyses, stratified by levels of PA, revealed that individuals in the Recommended and Additional groups experienced a lower risk of mortality compared with those in the None/Insufficient group; however, these differences were not statistically significant. Trend tests revealed a significant trend towards decreased mortality risk with escalating levels of PA (*p* for trend = 0.0003). All 3e Cox models (Models 1–3) satisfied the proportional hazards assumption. The Schoenfeld residual tests for proportionality were non-significant for each model (global tests: all *p* > 0.05), and no covariate-specific test indicated any violation of the assumption.

**Table II:** Association of physical activity with all-cause mortality

Item	Adjusted hazard ratio (95% confidence interval)^a^ per SD^b^, *p*-value		
	Model 1	Model 2	Model 3
^c^MET-h/week	0.73 (0.58, 0.92) 0.0083	0.78 (0.62, 0.97) 0.0257	0.88 (0.79, 0.99) 0.0169
None/Insufficient (0.00–7.49 ^c^MET-h/week)	Reference	Reference	Reference
Recommended (7.50–15.00 MET h/week)	0.80 (0.27, 2.35) 0.6800	0.82 (0.28, 2.39) 0.7138	0.87 (0.30, 2.51) 0.7989
Additional (>15 MET h/week)	0.37 (0.12, 1.10) 0.0740	0.42 (0.14, 1.26) 0.1224	0.43 (0.15, 1.26) 0.1248
Trend test	0.0002	0.0017	0.0003

Model 1: adjusted for age, gender. Model 2: adjusted for age, gender, education, gross annual income, marital status, tea consumption, tobacco consumption, alcohol consumption, body mass index (BMI), body fat percentage, body fat mass, muscle mass, muscle rate percentage, visceral adiposity index. Model 3: adjusted for age, gender, education, gross annual income, marital status, tea consumption, tobacco consumption, alcohol consumption, BMI, body fat percentage, body fat mass, muscle mass, muscle rate percentage, visceral adiposity index, lung function, TB, chronic bronchitis, asthma, diabetes, hypertension, CKD, RA, blood glucose, TG, TC, LDL, HDL.

a Cox proportional hazards models were used to estimate HRs and 95% 95% CIs.

b Log-transformed metabolic equivalent (MET)-h/week distributions were standardized to mean 0 and standard deviation [SD] 1, to facilitate comparison of effect sizes between biomarkers.

c Fitting model by standard Cox proportional hazards model.

### Threshold effect analysis of PA on all-cause mortality in all the participants

To investigate the threshold effect of PA on all-cause mortality, a Cox proportional hazards regression model integrated with a generalized additive model and smooth curve fitting (employing the penaliszed spline method) was utilized. The resulting smooth curve fitting revealed a non-linear “L”-shaped association between PA and all-cause mortality (Fig. S1). To further analyse this relationship, both a standard Cox proportional hazards model and a 2-piecewise Cox proportional hazards model were applied to examine the association between MET·h and all-cause mortality (Table III and Table SII). The log-likelihood ratio test yielded a *p*-value = 0.016, suggesting that the 2-piecewise Cox proportional hazards model better represented the relationship between PA and mortality. As depicted in Table III, there was a significant threshold effect, with the turning point occurring at 38.65 MET·h/week (95% CI: 26.76–74.59). For participants engaging in less than 38.65 MET·h/week, an increase in PA was associated with a notable reduction in the risk of all-cause mortality (HR = 0.58; 95% CI: 0.38–0.88; *p* = 0.0100). However, for those exceeding 38.65 MET·h/week, the association between PA and all-cause mortality became less pronounced (HR = 0.98; 95% CI: 0.96–1.01; *p* = 0.1782). Both the standard and 2-piecewise Cox models met the proportional-hazards assumption, evidenced by non-significant global Schoenfeld tests (*p* > 0.05) and no covariate-specific time-dependent effects.

**Table III:** Threshold effect analysis of physical activity on all-cause mortality in all the participants

Physical activity	Adjusted HR (95% CI)^a^ per SD^b^, *p*-value		
	Model 1	Model 2	Model 3
Turning point^c^		38.65 (26.76, 74.59)	
MET-h/week 38.65	0.48 (0.31, 0.74) 0.0010	0.55 (0.36, 0.83) 0.0042	0.58 (0.38, 0.88) 0.0100
MET-h/week ≥38.65	0.99 (0.96, 1.01) 0.1570	0.98 (0.95, 1.00) 0.0954	0.98 (0.96, 1.01) 0.1782
Likelihood ratio test *p*-value	0.002	0.009	0.016

Model 1: adjusted for age, gender. Model 2: adjusted for age, gender, education, gross annual income, marital status, tea consumption, tobacco consumption, alcohol consumption, BMI, body fat percentage, body fat mass, muscle mass, muscle rate percentage, visceral adiposity index. Model 3: adjusted for age, gender, education, gross annual income, marital status, tea consumption, tobacco consumption, alcohol consumption, BMI, body fat percentage, body fat mass, muscle mass, muscle rate percentage, visceral adiposity index, lung function, TB, chronic bronchitis, asthma, diabetes, hypertension, CKD, RA, blood glucose, TG, TC, LDL, HDL.

CI: confidence interval; HR: hazard ratio; MET: metabolic equivalent; SD: standard deviation.

a Cox proportional hazards models were used to estimate HRs and 95% 95% CIs.

b Log-transformed MET-h/week distributions were standardized to mean 0 and standard deviation [SD] 1, to facilitate comparison of effect sizes between biomarkers.

c We used a 2-piecewise logistic regression model with smoothing to analyse the association threshold between physical activity levels and all-cause mortality after adjusting the variables. The likelihood-ratio test and the bootstrap resampling method were used in determining inflection points.

### Association analysis between PA and all-cause mortality, stratified by airflow limitation severity

**Fig. 1:**
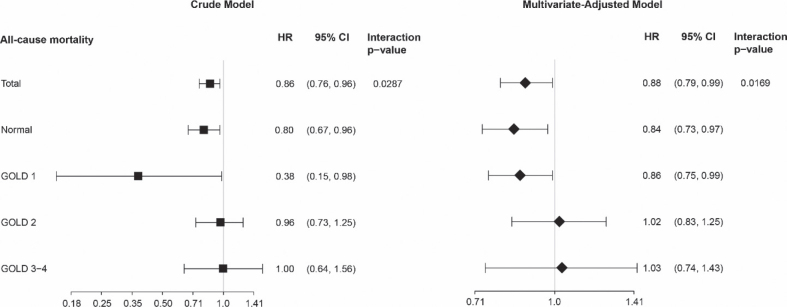
Association analysis between physical activity and all-cause mortality, stratified by different degrees of airflow restriction. Each stratification adjusted for all the factors (age, gender, education, gross annual income, marital status, tea consumption, tobacco consumption, alcohol consumption, BMI, body fat percentage, body fat mass, muscle mass, muscle rate percentage, visceral adiposity index, lung function, TB, chronic bronchitis, asthma, diabetes, hypertension, CKD, RA, blood glucose, TG, TC, LDL, HDL), except the stratification factor itself. Cl: confidence interval; HR: hazard ratio; GOLD: Global Initiative for Chronic Obstructive Lung Disease.

To elucidate the impact of airflow limitation on the relationship between PA and all-cause mortality, stratified analyses and interaction tests were performed according to varying degrees of airflow limitation. Utilizing a Cox proportional hazards model, we investigated the threshold effect of PA, measured as MET·h/week, on all-cause mortality within each stratum (Fig. 1). The findings revealed a significant inverse association between increased PA and mortality risk in the normal and GOLD 1 categories (HR = 0.84; 95% CI: 0.73–0.97; *p* = 0.0181, HR = 0.86; 95% CI: 0.75–0.99; *p* = 0.0317, respectively). Conversely, no significant associations were identified in the GOLD 2 and GOLD 3–4 categories (HR = 1.02; 95% CI: 0.83–1.25; *p* = 0.8670, HR = 1.03; 95% CI: 0.74–1.43; *p* = 0.8817, respectively). Schoenfeld residual analyses revealed no significant departures from the proportional-hazards assumption in any airflow-limitation stratum (*p* > 0.05). Additionally, a significant interaction was identified between airflow limitation and the relationship between PA and all-cause mortality (*p*-value for Interaction = 0.0169).

### Threshold effect analysis of PA on all-cause mortality in the participants with different degrees of airflow limitation

**Fig. 2:**
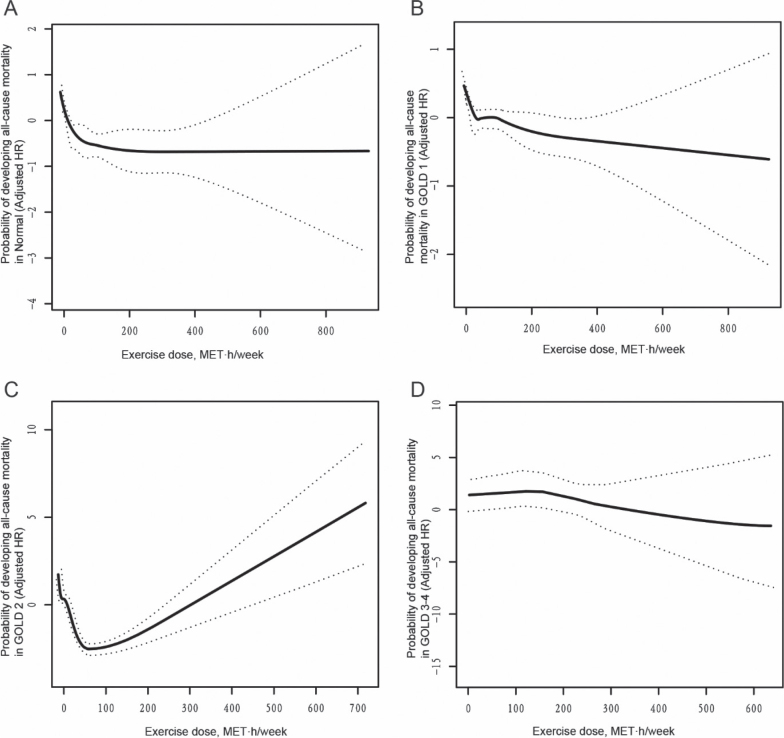
Association analysis between physical activity and all-cause mortality in the participants with different degrees of airflow restriction. A non-linear association between physical activity and all-cause mortality was found in the participants with different degrees of airflow restriction in a generalized additive model. The solid line and dashed line represent the estimated values and their corresponding 95% confidence intervals. Adjustment factors included age, gender, education, gross annual income, marital status, tea consumption, tobacco consumption, alcohol consumption, BMI, body fat percentage, body fat mass, muscle mass, muscle rate percentage, visceral adiposity index, lung function, TB, chronic bronchitis, asthma, diabetes, hypertension, CKD, RA, blood glucose, TG, TC, LDL, HDL. MET: metabolic equivalent; GOLD: Global Initiative for Chronic Obstructive Lung Disease.

We investigated the threshold effect of PA on all-cause mortality in participants with varying degrees of airflow limitation using a Cox proportional hazards model with penalized splines for smooth curve fitting. Results showed an “L”-shaped association in Normal and GOLD 1 subgroups, and a “U”-shaped association in the GOLD 2 subgroup; no significant associations were found in GOLD 3–4 subgroups (Fig. 2). In both the Normal and GOLD 1 subgroups, PA levels below the identified thresholds were associated with a significant reduction in all-cause mortality risk. For the Normal subgroup, the hazard ratio (HR) was 0.66 (95% CI: 0.52–0.84; *p* = 0.0006), and for GOLD 1, the HR was 0.41 (95% CI: 0.26–0.63; *p* < 0.0001). The turning points were MET·h/week = 41.50 (95% CI: 23.03–64.22) for Normal and MET·h/week = 13.21 (95% CI: 9.67–16.14) for GOLD 1. In the GOLD 2 subgroup, increased activity below the threshold reduced mortality risk (HR = 0.85; 95% CI: 0.81–0.90; *p* < 0.0001), but increased risk above it (HR = 1.23; 95% CI: 1.19–1.28; *p* < 0.0001) (Table IV and Table SIII). None of the Schoenfeld residual tests for the stratified models indicated any significant deviation from proportionality in any airflow limitation category (*p* > 0.05).

**Table IV:** Threshold effect analysis of physical activity on all-cause mortality in the participants with different degrees of airflow restriction

Physical activity	Adjusted HR (95% CI)^a^ per SD^b^, *p*-value		
	Model 1	Model 2	Model 3
Normal			
Turning point^c^		41.50 (23.03, 64.22)	
MET-h/week41.50	0.70 (0.54, 0.92) 0.0109	0.63 (0.48, 0.82) 0.0005	0.66 (0.52, 0.84) 0.0006
MET-h/week ≥ 41.50	0.99 (0.97, 1.01) 0.2738	0.99 (0.97, 1.01) 0.2754	0.99 (0.97, 1.01) 0.2942
Likelihood ratio test *p*-value	0.019	0.001	0.003
GOLD 1			
Turning point		13.21 (9.67, 16.14)	
MET-h/week13.21	0.49 (0.33, 0.73) 0.0004	0.23 (0.11, 0.49) 0.0001	0.41 (0.26, 0.63) <0.0001
MET-h/week ≥ 13.21	0.98 (0.97, 1.01) 0.1453	0.98 (0.96, 1.00) 0.0986	0.99 (0.97, 1.01) 0.2101
Likelihood ratio test *p*-value	< 0.001	< 0.001	< 0.001
GOLD 2			
Turning point		63.42 (45.02, 72.67)	
MET-h/week63.42	0.88 (0.85, 0.92) < 0.0001	0.89 (0.83, 0.94) < 0.0001	0.85 (0.81, 0.90) < 0.0001
MET-h/week ≥ 63.42	1.31 (1.26, 1.36) < 0.0001	1.33 (1.22, 1.44) < 0.0001	1.23 (1.19, 1.28) < 0.0001
Likelihood ratio test *p*-value	< 0.001	0.002	< 0.001
GOLD 3-4			
Turning point		173.68 (135.44, 241.27)	
MET-h/week173.68	1.23 (0.77, 1.98) 0.3887	—	—
MET-h/week ≥ 173.68	0.99 (0.95, 1.03) 0.6337	—	—
Likelihood ratio test *p*-value	0.371	—	—

Model 1: adjusted for age, gender. Model 2: adjusted for age, gender, education, gross annual income, marital status, tea consumption, tobacco consumption, alcohol consumption, BMI, body fat percentage, body fat mass, muscle mass, muscle rate percentage, visceral adiposity index. Model 3: adjusted for age, gender, education, gross annual income, marital status, tea consumption, tobacco consumption, alcohol consumption, BMI, body fat percentage, body fat mass, muscle mass, muscle rate percentage, visceral adiposity index, lung function, TB, chronic bronchitis, asthma, diabetes, hypertension, CKD, RA, blood glucose, TG, TC, LDL, HDL.

a Cox proportional hazards models were used to estimate hazard ratios (HRs) and 95% confidence intervals (CIs).

b Log-transformed metabolic equivalent (MET)-h/week distributions were standardized to mean 0 and standard deviation [SD] 1, to facilitate comparison of effect sizes between biomarkers.

c We used a 2-piecewise logistic regression model with smoothing to analyse the association threshold between physical activity levels and all-cause mortality after adjusting the variables. The likelihood-ratio test and the bootstrap resampling method were used in determining inflection points.

GOLD: Global Initiative for Chronic Obstructive Lung Disease.

### Sensitivity analysis

To evaluate the robustness of the relationship between PA and all-cause mortality among participants with varying degrees of airflow limitation, this study employed subgroup analyses coupled with interaction tests, as well as multiple imputation to handle missing covariate values. Subgroup analyses indicated that although differences were observed across some subgroups, the association between PA and all-cause mortality remained consistent despite potential confounders (Tables SIV–SVI). Due to limited sample sizes, further subgroup analyses could not be supported within the GOLD 2 and GOLD 3–4 groups. Additionally, the results from the dataset after multiple imputation were consistent with those from the original dataset, suggesting no substantial bias (Table SVII).

## DISCUSSION

This study elucidates the complex relationship between PA dose and mortality risk among individuals with varying severities of airflow limitation, a pivotal consideration in the management of COPD. Using a prospective cohort design supplemented by multivariate Cox proportional hazards modelling, penalized spline smoothing, and piecewise Cox models, our analysis revealed distinct threshold effects that demarcate beneficial from potentially harmful levels of PA. Specifically, for individuals without airflow limitation and those with mild impairment (GOLD 1), PA up to 41.50 and 13.21 MET·h/week, respectively, was associated with a significant reduction in all-cause mortality. In contrast, for patients with moderate airflow limitation (GOLD 2), higher activity levels above these thresholds correlated with an increased mortality risk, highlighting the importance of cautious prescription of intense exercise programmes. No significant associations were observed in severe to very severe cases (GOLD 3–4).

A meta-analysis corroborates previous findings that early pulmonary rehabilitation reduces both short-term and long-term mortality rates when compared with control groups (24). Another meta-analysis revealed that early and late pulmonary rehabilitation initiated within and after 1 week, respectively, led to decreased rehospitalization rates over 3 to 6 months; however, no significant differences were observed in mortality rates after 1 year (25). Despite the short-term benefits of early rehabilitation, further investigation is required to elucidate its long-term efficacy. Moreover, existing literature indicates that PA enhances exercise capacity, improves quality of life, and alleviates respiratory symptoms in patients with COPD (26, 27). Nevertheless, the relationship between PA and mortality in COPD appears less pronounced than in other conditions such as T2D, IHD, and breast cancer (14). This discrepancy may be attributed to several factors, including the limitations of employing linear models for analysis, which do not account for potential non-linear relationships or threshold effects. Our study used a Cox proportional hazards model integrated with a generalized additive model and smooth curve fitting via penalized splines to reveal a U-shaped association between PA intensity and mortality risk, suggesting that linear models might mask underlying non-linear relationships. Indeed, evidence from endurance athletes and general population cohorts suggests that very high exercise volumes are associated with certain health risks (e.g., myocardial fibrosis, atrial fibrillation, and sudden cardiac events), supporting the concept that “more is not always better” beyond an optimal activity range (28). This pattern aligns with the so-called physical-activity paradox in occupational epidemiology, where vigorous job-related exertion, unlike leisure-time exercise, correlates with increased health risk (29). Thus, it is biologically plausible that in individuals with moderate COPD there exists an optimal exercise dose, below or above which the benefits wane and potential harms emerge.

Furthermore, our analysis demonstrates that different levels of airflow obstruction are associated with distinct thresholds of PA where benefits or risks may vary. For instance, the turning points were identified at 41.50 MET·h/week (95% CI: 23.03–64.22) for healthy individuals and 13.21 MET·h/week (95% CI: 9.67–16.14) for patients classified as GOLD 1. This indicates that patients with mild COPD (GOLD 1) may benefit from lower volumes of exercise compared with healthy controls. Conversely, in patients categorized as GOLD 2, PA exceeding a specific threshold may be deleterious, potentially due to increased oxygen consumption, cardiac load, and oxidative stress, exacerbating underlying disease (30–34). Moreover, monocytes/macrophages serve as the frontline defence in immune responses against pathogens. These cells play pivotal roles in antigen presentation, chemotaxis, phagocytosis, microbicidal activities, tumour killing, and secretion functions, contributing to innate immunity by initiating inflammatory and immune responses. Intense physical exertion might impair macrophage function, leading to pulmonary immunosuppression and increasing susceptibility to infections (35–37).

In line with the Global Initiative for Chronic Obstructive Lung Disease (GOLD) guidelines, which categorize airflow limitation severity into 4 stages (38–40), our study provides new insights into the differential effects of exercise interventions across these stages. We employed subgroup analyses and interaction tests to demonstrate that the relationship between PA and mortality varies significantly according to the degree of airflow limitation. These findings support the need for personalized exercise regimens tailored to individual patient characteristics. In summary, our research highlights the importance of considering non-linear and threshold effects in the relationship between PA and mortality risk in COPD patients.

Differences in the association between physical activity and mortality across COPD severity levels may be explained by distinct physiological adaptations and stress responses at each GOLD stage. Patients with mild COPD have relatively preserved cardiopulmonary reserve and less skeletal muscle dysfunction, so regular physical activity can improve cardiovascular fitness and muscular efficiency, which helps lower systemic inflammation and enhances survival (41). In contrast, advanced COPD is characterized by chronic inactivity and pronounced muscle atrophy (with a shift from oxidative Type I to glycolytic Type II fibres) that reduces exercise tolerance and blunts the beneficial effects of exercise (41, 42). While moderate exercise in these patients still boosts mitochondrial function and oxidative capacity in muscle (raising the threshold for lactic acid buildup), excessively intense activity may acutely provoke heightened oxidative stress, sympathetic activation, and even transient immune suppression (43), potentially negating benefits. Moreover, individuals with more severe airflow limitation experience greater dynamic lung hyperinflation and hypoxemia during exertion, which strain the cardiovascular system and can elevate arrhythmia or exacerbation risk (44). These factors may explain why higher doses of physical activity confer diminishing or even adverse returns in moderate-to-severe COPD, underscoring the need for stage-tailored exercise prescriptions that maximize benefits while avoiding physiological harm (45).

Notwithstanding the strengths of our study, it is important to acknowledge certain limitations. First, our study primarily focused on all-cause mortality as the outcome measure, omitting specific information on chronic respiratory disease-related mortalities. This gap precludes a more detailed examination of how varying degrees of airflow limitation influence PA and other cause-specific mortality associations. Second, due to the relatively small sample size, particularly within the GOLD 2 and GOLD 3–4 subgroups, we were constrained in performing comprehensive subgroup analyses and interaction tests. Consequently, our findings, especially regarding the relationship between PA and all-cause mortality in GOLD 3–4 patients, require validation through larger, prospective cohort studies. Third, the study cohort was drawn from a single rural region in Northwest China, which may limit the generalizability of our findings to other populations in different geographic or urban settings. Lastly, our investigation centred on the total amount of PA as a quantitative measure, neglecting other dimensions such as frequency, type, and duration of exercise, which have been extensively studied in relation to airflow limitation severity. Therefore, a more holistic understanding of how exercise rehabilitation impacts mortality across different levels of airflow limitation remains to be elucidated.

In conclusion, the present prospective cohort study elucidates the nuanced relationship between PA dosage and mortality risk across varying severities of airflow limitation. Analysing data from the prospective cohort study reveals distinct threshold effects that underscore the need for tailored exercise prescriptions. These results provide valuable insights into the dose–response dynamics of PA and suggest that a one-size-fits-all approach to exercise rehabilitation may not be appropriate for COPD patients. Instead, personalized exercise regimens based on the severity of airflow limitation could potentially optimize health outcomes and reduce mortality risk. Future research should focus on validating these findings in diverse populations and provide insights for clinicians to develop and implement personalized exercise programmes.

## Supplementary Material





## Data Availability

As one of the key funded projects, “Natural Population Cohort Study in Northwest China” (SQ2017YFSF090013) aims to study the aetiology and disease burden of characteristic chronic diseases in Northwest China and to analyse the relationship between chronic diseases and health in northwest China, including natural environment and behavioural patterns. “Study on cohort Construction and Health Follow-up of Xinjiang Multi-ethnic natural Population” (2017YFC0907203) is a sub-project of this project. Our study population are all from this project. The datasets used and/or analysed during the present study are available from the corresponding author on reasonable request.
