# Supplementary material for: DIFFERENTIAL PATTERNS OF THE RELATIONSHIP BETWEEN EXERCISE DOSE AND MORTALITY RISK ACROSS SEVERITIES OF AIRFLOW LIMITATION: A PROSPECTIVE COHORT STUDY WITH A 5-YEAR FOLLOW-UP PERIOD
Source: J Rehabil Med. 2025 Jun 16;57:43377. doi: 10.2340/jrm.v57.43377 (PMC12186442; doi:10.2340/jrm.v57.43377)
Supplement: Supplementary file 1 [file JRM-57-43377-s1.pdf]

Supplementary material has been published as submitted. It has not been copyedited, or typeset by Journal of Rehabilitation Medicine

Table SI. Characteristics and Baseline Results of Participants Stratified by Gender.

Characteristics		Total (N=2968)	Male（N=1495）	Female（N=1473）	P-value
Age, y*		51.47 ± 9.80	53.21 ± 10.24	49.71 ± 9.00	<0.001
Education, %***					<0.001
	Illiterate	14.28	13.53	15.04	
	Primary school	71.17	67.38	75.02	
	Junior high school	12.80	16.34	9.19	
	High school and above	1.76	2.75	0.75	
Gross annual income, yuan***					<0.001
	<10,000	54.23	48.99	59.54	
	≥10,000	45.77	51.01	40.46	
Marital status***					<0.001
	Married	85.01	90.98	78.93	
	Divorce and so on	14.99	9.02	21.07	
Tea consumption***					0.012
	Yes	52.23	49.93	54.56	
	No	47.77	50.07	45.44	
Tobacco consumption***					<0.001
	Yes	13.52	26.12	0.75	
	No	86.48	73.88	99.25	
Alcohol consumption***					<0.001

	Yes	9.31	17.72	0.82	
	No	90.69	82.28	99.18	
[a]BMI, kg/m2*		26.93 ± 6.17	26.07 ± 4.19	27.78 ± 7.55	<0.001
Body fat percentage, %*		31.48 ± 9.50	25.07 ± 6.19	38.00 ± 7.69	<0.001
Body fat mass, kg*		20.87 ± 8.50	17.60 ± 6.87	24.21 ± 8.72	<0.001
Muscle mass, kg*		41.84 ± 8.29	47.94 ± 6.45	35.65 ± 4.51	<0.001
muscle rate percentage, %*		0.65 ± 0.09	0.71 ± 0.06	0.59 ± 0.08	<0.001
visceral adiposity index*		10.34 ± 4.30	12.92 ± 3.95	7.73 ± 2.81	<0.001
Physical activity, [b]MET· h/week**		45.16 ± 73.71	44.57 ± 71.23	45.38 ± 75.17	0.802
FVC% predicted*		96.69 ± 19.36	97.33 ± 19.12	96.02 ± 19.61	0.064
FEV1% predicted*		88.45 ± 20.35	89.30 ± 20.17	87.64 ± 20.54	0.026
FEV1/FVC*		76.11 ± 9.66	75.31 ± 10.29	76.98 ± 8.90	<0.001
TB***					0.009
	No	95.02	93.96	96.13	
	Yes	4.98	6.04	3.87	
COPD***					0.145
	No	78.76	77.86	80.04	
	Yes	21.24	22.14	19.96	
Chronic bronchitis***					<0.001
	No	83.33	86.56	79.97	
	Yes	16.67	13.44	20.03	

Asthma***					<0.001
	No	97.21	98.26	96.13	
	Yes	2.79	1.74	3.87	
Diabetes***					0.746
	No	96.17	96.29	96.05	
	Yes	3.83	3.71	3.95	
Hypertension***					0.211
	No	69.32	70.48	68.21	
	Yes	30.68	29.52	31.79	
CKD***					0.001
	No	92.97	91.44	94.50	
	Yes	7.03	8.56	5.50	
RA***					<0.001
	No	86.15	88.83	83.37	
	Yes	13.85	11.17	16.63	
Blood glucose, mmol/L*		5.33 ± 1.49	5.37 ± 1.60	5.30 ± 1.37	0.503
TG, mmol/L**		1.43 ± 1.16	1.47 ± 1.29	1.40 ± 1.01	0.950
TC, mmol/L*		4.63 ± 1.36	4.54 ± 1.36	4.72 ± 1.34	<0.001
LDL, mmol/L*		2.58 ± 0.78	2.59 ± 0.76	2.57 ± 0.80	0.197
HDL, mmol/L*		1.32 ± 0.48	1.29 ± 0.50	1.36 ± 0.46	<0.001

Note: For continuous variables, inter-group differences were assessed with Student's t-test* when the data conformed to a normal distribution; when normality was not satisfied, the Mann–Whitney U test** was employed. Comparisons of categorical variables between groups were conducted using χ^2 tests***.

Abbreviations: BMI, body mass index; MET, Metabolic Equivalent; FEV1, forced expiratory volume in 1 s; FVC, forced vital capacity; TB, Tuberculosis; COPD, Chronic obstructive pulmonary; disease; CKD chronic kidney disease; RA, Rheumatoid arthritis; TG, Triglyceride; TC, Total Cholesterol; LDL, Low Density Lipoprotein; HDL High Density Lipoprotein.

[a]BMI was calculated as the body weight in kilograms divided by the square of the height in meters.

[b]MET· h=MET score × exercise time.

Table SII. Threshold Effect Analysis of physical activity on All-Cause Mortality in all the participants (Without Log Transformation of MET-h/week).

Physical activity	[a]Adjusted HR (95% CI), P Value		
	Model 1	Model 2	Model 3
[b]Turning point	38.65 (26.76, 74.59)		
MET-h/week＜38.65	0.91 (0.86, 0.97) 0.0010	0.93 (0.86, 0.99) 0.0042	0.95 (0.91, 0.99) 0.0100
MET-h/week≥38.65	1.00 (1.00, 1.00) 0.1570	1.00 (1.00, 1.00) 0.0954	1.00 (1.00, 1.00) 0.1782
Likelihood ratio test p	0.002	0.009	0.016

Note: Statistical analysis was conducted using the raw data of MET-h/week, without applying logarithmic transformation.

Model 1: adjusted for Age, Gender.

Model 2: adjusted for Age, Gender, Education, Gross annual income, Marital status, Tea consumption, Tobacco consumption, Alcohol consumption, BMI, Body fat percentage, Body fat mass, Muscle mass, muscle rate percentage, visceral adiposity index.

Model 3: adjusted for Age, Gender, Education, Gross annual income, Marital status, Tea consumption, Tobacco consumption, Alcohol consumption, BMI, Body fat percentage, Body fat mass, Muscle mass, muscle rate percentage, visceral adiposity index, Lung function, TB, Chronic bronchitis， Asthma, Diabetes, Hypertension, CKD, RA, Blood glucose, TG, TC, LDL, HDL.

Abbreviations: CI, confidence interval; HR, hazard ratio; MET, Metabolic Equivalent; SD, Standard deviation.

[a]Cox proportional hazards models were used to estimate HRs and 95% 95% CIs.

[b]We used a two-piece-wise logistic regression model with smoothing to analyze the association threshold between physical activity levels and All-Cause Mortality after adjusting the variables. The likelihood-ratio test and the bootstrap resampling method were used in determining inflection points.

Table SIII. Threshold Effect Analysis of physical activity on All-Cause Mortality in the participants with different degrees of airflow restriction (Without Log Transformation of MET-h/week).

Physical activity		[a]Adjusted HR (95% CI), P Value		
		Model 1	Model 2	Model 3
Normal				
	[b]Turning point	41.50 (23.03, 64.22)		
	MET-h/week＜41.50	0.96 (0.94, 0.99) 0.0109	0.95 (0.92, 0.98) 0.0005	0.96 (0.92, 0.99) 0.0006
	MET-h/week≥41.50	1.00 (1.00, 1.00) 0.2738	1.00 (1.00, 1.00) 0.2754	1.00 (1.00, 1.00) 0.2942
	Likelihood ratio test p	0.019	0.001	0.003
GOLD 1				
	Turning point	13.21 (9.67, 16.14)		
	MET-h/week＜13.21	0.94 (0.89, 0.99) 0.0004	0.89 (0.81, 0.96) 0.0001	0.92 (0.87, 0.97) <0.0001
	MET-h/week≥13.21	1.00 (1.00, 1.00) 0.1453	1.00 (1.00, 1.00) 0.0986	1.00 (1.00, 1.00) 0.2101
	Likelihood ratio test p	<0.001	<0.001	<0.001
GOLD 2				
	Turning point	63.42 (45.02, 72.67)		
	MET-h/week＜63.42	0.98 (0.96, 1.00) <0.0001	0.98 (0.96, 1.00) <0.0001	0.98 (0.97, 1.00) <0.0001
	MET-h/week≥63.42	1.02 (1.01, 1.03) <0.0001	1.02 (1.01, 1.03) <0.0001	1.01 (1.01, 1.02) <0.0001
	Likelihood ratio test p	<0.001	0.002	<0.001
GOLD 3-4				
	Turning point	173.68 (135.44, 241.27)		
	MET-h/week＜173.68	1.01 (0.98, 1.04) 0.3887	–	–
	MET-h/week≥173.68	1.00 (1.00, 1.00) 0.6337	–	–

Likelihood ratio test p	0.371	–	–

Note: Statistical analysis was conducted using the raw data of MET-h/week, without applying logarithmic transformation.

Model 1: adjusted for Age, Gender.

Model 2: adjusted for Age, Gender, Education, Gross annual income, Marital status, Tea consumption, Tobacco consumption, Alcohol consumption, BMI, Body fat percentage, Body fat mass, Muscle mass, muscle rate percentage, visceral adiposity index.

Model 3: adjusted for Age, Gender, Education, Gross annual income, Marital status, Tea consumption, Tobacco consumption, Alcohol consumption, BMI, Body fat percentage, Body fat mass, Muscle mass, muscle rate percentage, visceral adiposity index, Lung function, TB, Chronic bronchitis， Asthma, Diabetes, Hypertension, CKD, RA, Blood glucose, TG, TC, LDL, HDL.

Abbreviations: CI, confidence interval; HR, hazard ratio; MET, Metabolic Equivalent; SD, Standard deviation, GOLD, Global Initiative for Chronic Obstructive Lung Disease.

[a]Cox proportional hazards models were used to estimate HRs and 95% 95% CIs.

[b]We used a two-piece-wise logistic regression model with smoothing to analyze the association threshold between physical activity levels and All-Cause Mortality after adjusting the variables. The likelihood-ratio test and the bootstrap resampling method were used in determining inflection points.

Table SIV. Stratified analysis for Relationship of physical activity with All-Cause Mortality in various subgroups divided at 41.50 (MET-h/week＜41.50, MET-h/week≥41.50) in normal group.

	MET-h/week＜41.50			MET-h/week≥41.50		
Characteristics	[a]Adjusted HR (95% CI)	P value	P for interaction	[a]Adjusted HR (95% CI)	P value	P for interaction
Age, y						
＜65	0.53 (0.32, 0.88)	0.0136	0.1422	1.00 (0.98, 1.01)	0.7178	0.6968
≥65	0.64 (0.36, 1.14)	0.1288		1.00 (0.99, 1.02)	0.6638	
Gender						
Male	0.73 (0.55, 0.96)	0.0236	0.1705	1.00 (0.98, 1.02)	0.7881	0.8991
Female	0.83 (0.61, 1.13)	0.2343		1.00 (0.99, 1.02)	0.6780	
Education, %						
Primary school and below	0.73 (0.51, 1.03)	0.0725	0.5377	1.00 (0.98, 1.01)	0.5817	0.7934
Junior high school and above	0.74 (0.57, 0.95)	0.0169		1.00 (0.99, 1.01)	0.9319	
Gross annual income, yuan						
< 10,000	0.65 (0.37, 1.14)	0.1348	0.8545	1.00 (0.99, 1.02)	0.6648	0.2348
≥ 10,000	0.54 (0.34, 0.84)	0.0066		1.00 (0.98, 1.01)	0.7161	
Marital status						
Married	0.40 (0.19, 0.86)	0.0185	0.1779	0.99 (0.98, 1.01)	0.4052	0.4894
Divorce and so on	0.52 (0.21, 1.28)	0.1546		1.00 (0.99, 1.02)	0.7961	
TB						
No	0.79 (0.54, 1.15)	0.2164	0.1479	1.00 (0.99, 1.02)	0.4616	0.7994
Yes	0.72 (0.49, 1.05)	0.0911		1.00 (0.98, 1.02)	0.9389	

Chronic bronchitis						
No	0.83 (0.67, 1.02)	0.0729	0.1551	1.00 (0.99, 1.01)	0.6643	0.6930
Yes	0.79 (0.58, 1.07)	0.1225		1.00 (0.98, 1.02)	0.8954	
Asthma						
No	0.69 (0.51, 0.93)	0.0148	0.3906	1.00 (0.99, 1.01)	0.8915	0.1400
Yes	0.67 (0.44, 1.02)	0.0641		0.99 (0.97, 1.02)	0.5845	
Diabetes						
No	0.78 (0.60, 1.01)	0.0575	0.2830	1.00 (0.99, 1.01)	0.5427	0.6405
Yes	0.75 (0.47, 1.18)	0.2139		1.00 (0.99, 1.02)	0.5300	
Hypertension						
No	0.37 (0.13, 1.07)	0.0663	0.3635	0.99 (0.97, 1.01)	0.4004	0.4741
Yes	0.44 (0.15, 1.33)	0.1459		1.00 (0.99, 1.02)	0.6046	
CKD						
No	0.51 (0.21, 1.26)	0.1444	0.8315	0.99 (0.97, 1.01)	0.3894	0.2521
Yes	0.55 (0.18, 1.64)	0.2831		1.00 (0.99, 1.02)	0.7516	
RA						
No	0.57 (0.32, 1.02)	0.0596	0.1945	1.00 (0.99, 1.01)	0.8618	0.7838
Yes	0.50 (0.22, 1.14)	0.1002		1.00 (0.98, 1.02)	0.8834	

Abbreviations: MET, Metabolic Equivalent; TB, Tuberculosis; COPD, Chronic obstructive pulmonary; disease; CKD chronic kidney disease; RA, Rheumatoid arthritis.

[a]Adjusted for all variables except those required for stratification.

[b]MET· h=MET score × exercise time.

Table SV. Stratified analysis for Relationship of physical activity with All-Cause Mortality in various subgroups divided at 13.21 (MET-h/week＜13.21, MET-h/week≥13.21) in GOLD 1.

Characteristics	MET-h/week＜13.21			MET-h/week≥13.21		
	[a]Adjusted HR (95% CI)	P value	P for interaction	[a]Adjusted HR (95% CI)	P value	P for interaction
Age, y						
＜60	0.64 (0.45, 0.90)	0.0114	0.2196	1.00 (0.99, 1.01)	0.9979	0.4917
≥60	0.62 (0.37, 1.04)	0.0716		1.00 (0.99, 1.02)	0.4677	
Gender						
Male	0.80 (0.61, 1.04)	0.0984	0.5258	1.00 (0.98, 1.02)	0.9118	0.7816
Female	0.81 (0.67, 0.98)	0.0288		1.00 (0.99, 1.01)	0.8229	
Education, %						
Primary school and below	0.78 (0.57, 1.05)	0.1021	0.0827	1.00 (0.99, 1.02)	0.6079	0.6175
Junior high school and above	0.75 (0.57, 1.00)	0.0534		1.00 (0.98, 1.01)	0.5895	
Gross annual income, yuan						
< 10,000	0.82 (0.59, 1.14)	0.2357	0.0760	1.00 (0.99, 1.02)	0.6752	0.2797
≥ 10,000	0.71 (0.53, 0.95)	0.0208		1.00 (0.98, 1.02)	0.8576	
Marital status						
Married	0.77 (0.62, 0.94)	0.0113	0.1409	1.00 (0.99, 1.01)	0.9404	0.8864
Divorce and so on	0.81 (0.61, 1.07)	0.1357		1.00 (0.97, 1.02)	0.7312	
TB						
No	0.67 (0.42, 1.08)	0.0990	0.2655	1.01 (0.99, 1.02)	0.3731	0.5434
Yes	0.63 (0.39, 1.02)	0.0628		0.99 (0.98, 1.01)	0.5314	

Chronic bronchitis						
No	0.79 (0.64, 0.97)	0.0235	0.7845	1.00 (0.99, 1.01)	0.8475	0.3294
Yes	0.77 (0.57, 1.04)	0.0923		1.01 (0.99, 1.02)	0.4011	
Asthma						
No	0.78 (0.57, 1.06)	0.1116	0.1007	1.00 (0.98, 1.01)	0.7696	0.2246
Yes	0.80 (0.58, 1.12)	0.1944		1.00 (0.99, 1.02)	0.5213	
Diabetes						
No	0.80 (0.64, 1.00)	0.0527	0.3050	1.00 (0.99, 1.01)	0.8201	0.7100
Yes	0.74 (0.59, 0.93)	0.0086		1.00 (0.99, 1.01)	0.9269	
Hypertension						
No	0.77 (0.59, 1.00)	0.0484	0.1027	0.99 (0.96, 1.01)	0.3477	0.3422
Yes	0.82 (0.61, 1.11)	0.1963		1.01 (0.99, 1.02)	0.5095	
CKD						
No	0.79 (0.65, 0.95)	0.0147	0.1276	1.00 (0.99, 1.01)	0.8140	0.3181
Yes	0.67 (0.44, 1.02)	0.0620		1.00 (0.98, 1.01)	0.5781	
RA						
No	0.70 (0.51, 0.96)	0.0265	0.0882	1.00 (0.98, 1.01)	0.7989	0.5400
Yes	0.76 (0.54, 1.07)	0.1221		1.00 (0.99, 1.02)	0.6404	

Abbreviations: MET, Metabolic Equivalent; TB, Tuberculosis; COPD, Chronic obstructive pulmonary; disease; CKD chronic kidney disease; RA, Rheumatoid arthritis; GOLD, Global Initiative for Chronic Obstructive Lung Disease.

[a]Adjusted for all variables except those required for stratification.

[b]MET· h=MET score × exercise time.

Table SVI. Stratified analysis for Relationship of physical activity with All-Cause Mortality in various subgroups divided at 63.42 (MET-h/week＜63.42, MET-h/week≥63.42) in GOLD 2.

	[a]MET-h/week＜63.42			MET-h/week≥63.42		
Characteristics	[b]Adjusted HR (95% CI)	P value	P for interaction	[b]Adjusted HR (95% CI)	P value	P for interaction
Age, y						
＜60	0.93 (0.80, 0.99)	0.0312	0.1200	1.30 (1.16, 1.46)	<0.0001	0.2999
≥60	0.87 (0.83, 0.92)	<0.0001		1.35 (1.28, 1.42)	<0.0001	
Gender						
Male	0.88 (0.84, 0.91)	<0.0001	0.1821	2.11 (1.93, 2.30)	<0.0001	0.4086
Female	0.93 (0.88, 0.98)	0.0086		1.70 (1.38, 2.11)	<0.0001	
Hypertension						
No	0.87 (0.80, 0.93)	0.0002	0.5717	0.00 (0.00, inf.)	0.1155	0.1491
Yes	0.85 (0.78, 0.92)	<0.0001		0.11 (0.00, 7.37)	0.3077	

Abbreviations: MET, Metabolic Equivalent; GOLD, Global Initiative for Chronic Obstructive Lung Disease.

[a]MET· h=MET score × exercise time.

[b]Adjusted for all variables except those required for stratification.

Table SVII. Threshold Effect Analysis of physical activity on All-Cause Mortality in the participants with different degrees of airflow restriction. (Multiple Imputation)

Physical activity		[a]Adjusted HR (95% CI) [b]per SD, P Value		
		Model 1	Model 2	Model 3
Normal				
	[C]Turning point	41.50 (23.03, 64.22)		
	MET-h/week < 41.50	0.68 (0.50, 0.92) 0.0130	0.60 (0.39, 0.91) 0.0165	0.65 (0.44, 0.96) 0.0299
	MET-h/week≥41.50	1.00 (0.99, 1.01) 0.9700	1.00 (0.99, 1.01) 0.9128	1.00 (0.99, 1.01) 0.8963
GOLD 1				
	Turning point	13.21 (9.67, 16.14)		
	MET-h/week < 13.21	0.50 (0.30, 0.81) 0.0055	0.33 (0.10, 1.06) 0.0637	0.44 (0.19, 1.03) 0.0585
	MET-h/week≥13.21	1.00 (0.99, 1.01) 0.9859	1.00 (0.98, 1.02) 0.8266	1.00 (0.99, 1.01) 0.7683
GOLD 2				
	Turning point	63.42 (45.02, 72.67)		
	MET-h/week < 63.42	0.86 (0.81, 0.91) <0.0001	0.87 (0.83, 0.90) <0.0001	0.84 (0.78, 0.92) <0.0001
	MET-h/week≥63.42	1.39 (1.33, 1.47) <0.0001	1.73 (1.62, 1.86) <0.0001	1.36 (1.24, 1.50) <0.0001
GOLD 3-4				
	Turning point	173.68 (135.44, 241.27)		
	MET-h/week < 173.68	1.01 (0.96, 1.06) 0.6735	–	–
	MET-h/week≥173.68	0.39 (0.07, 2.16) 0.2843	–	–

Model 1: adjusted for Age, Gender.

Model 2: adjusted for Age, Gender, Education, Gross annual income, Marital status, Tea consumption, Tobacco consumption, Alcohol consumption, BMI, Body fat percentage, Body fat mass, Muscle mass, muscle rate percentage, visceral adiposity index.

Model 3: adjusted for Age, Gender, Education, Gross annual income, Marital status, Tea consumption, Tobacco consumption, Alcohol consumption, BMI, Body fat percentage, Body fat mass, Muscle mass, muscle rate percentage, visceral adiposity index, Lung function, TB, Chronic bronchitis， Asthma, Diabetes, Hypertension, CKD, RA, Blood glucose, TG, TC, LDL, HDL.

Abbreviations: CI, confidence interval; HR, hazard ratio; MET, Metabolic Equivalent; SD, Standard deviation; GOLD, Global Initiative for Chronic Obstructive Lung Disease.

[a]Cox proportional hazards models were used to estimate HRs and 95% 95% CIs.

[b]Log-transformed MET-h/week distributions were standardized to mean 0 and standard deviation [SD] 1, to facilitate comparison of effect sizes between biomarkers.

[C]We used a two-piece-wise logistic regression model with smoothing to analyze the association threshold between physical activity levels and All-Cause Mortality after adjusting the variables. The likelihood-ratio test and the bootstrap resampling method were used in determining inflection points.
