# Supplementary material for: DIFFERENTIAL PATTERNS OF THE RELATIONSHIP BETWEEN EXERCISE DOSE AND MORTALITY RISK ACROSS SEVERITIES OF AIRFLOW LIMITATION: A PROSPECTIVE COHORT STUDY WITH A 5-YEAR FOLLOW-UP PERIOD
Source: J Rehabil Med. 2025 Jun 16;57:43377. doi: 10.2340/jrm.v57.43377 (PMC12186442; doi:10.2340/jrm.v57.43377)

Supplementary material has been published as submitted. It has not been copyedited, or typeset by Journal of Rehabilitation Medicine

Fig. S1. Association analysis between physical activity and All-Cause Mortality in all the participants.

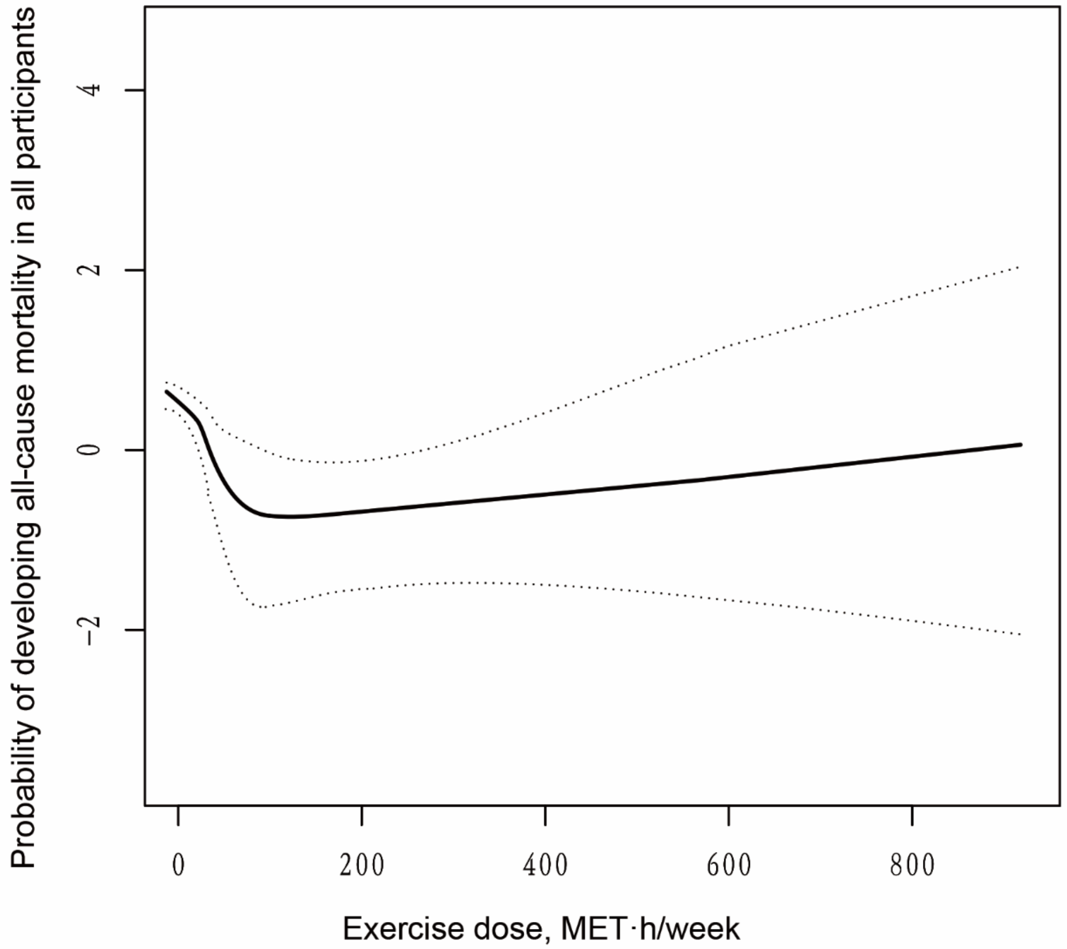

Supplement: Supplementary file 2 [file JRM-57-43377-s2.pdf]
